# Zinc and the Innovative Zinc-α2-Glycoprotein Adipokine Play an Important Role in Lipid Metabolism: A Critical Review

**DOI:** 10.3390/nu13062023

**Published:** 2021-06-11

**Authors:** Michalina Banaszak, Ilona Górna, Juliusz Przysławski

**Affiliations:** 1 Faculty of Medical Sciences, Poznan University of Medical Sciences, 60-812 Poznan, Poland; mi.banaszak97@gmail.com; 2 Department of Bromatology, Poznan University of Medical Sciences, 60-354 Poznan, Poland; jprzysla@ump.edu.pl

**Keywords:** zinc, zinc-α2-glycoprotein, lipid metabolism

## Abstract

Numerous studies indicate that zinc and the new zinc-related adipokine, zinc-α2-glycoprotein (ZAG), are involved in lipid metabolism. Excess body fat lowers blood concentrations of Zn and ZAG, leading not only to the development of obesity but also to other components of the metabolic syndrome. Zinc homeostasis disorders in the body negatively affect the lipid profile and cytokine secretion. Zinc appears to be a very important ZAG homeostasis regulator. The physiological effects of ZAG are related to lipid metabolism, but studies show that ZAG also affects glucose metabolism and is linked to insulin resistance. ZAG has a zinc binding site in its structure, which may indicate that ZAG mediates the effect of zinc on lipid metabolism. The review aimed to verify the available studies on the effects of zinc and ZAG on lipid metabolism. A literature review within the scope of this research area was conducted using articles available in PubMed (including MEDLINE), Web of Science and Cochrane Library databases. An analysis of available studies has shown that zinc improves hepatic lipid metabolism and has an impact on the lipid profile. Numerous studies have found that zinc supplementation in overweight individuals significantly reduced blood levels of total cholesterol, LDL (Low-density lipoprotein)cholesterol and triglycerides, potentially reducing cardiovascular morbidity and mortality. Some results also indicate that it increases HDL-C (High-density lipoprotein) cholesterol levels. ZAG has been shown to play a significant role in reducing obesity and improving insulin sensitivity, both in experimental animal model studies and in human studies. Furthermore, ZAG at physiologically relevant concentrations increases the release of adiponectin from human adipocytes. In addition, ZAG has been shown to inhibit in vitro leptin production. Further studies are needed to provide more data on the role of zinc and zinc-α2-glycoprotein.

## 1. Introduction

Zinc, as a trace element, plays an important role in human life. It is involved in the growth and development of the body and, as a catalyst for enzymes, participates in the metabolism of lipids, proteins and carbohydrates. It regulates the expression and activation of many molecules and builds over three hundred metalloenzymes. This metal is involved in the regulation of inflammations by affecting pro-inflammatory cytokines and plays a role in reducing oxidative stress. Zinc deficiency impairs growth, reproduction, metabolism and immunity in both humans and animals and may predispose those affected to the development of obesity and other metabolic diseases [1,2,3,4]. As obesity is now a modern-age disease, attempts are being made to find factors responsible for causing it so that it can be treated effectively [5]. In addition to zinc, zinc-α2-glycoprotein (ZAG) also appears to be important for the human body, as it plays an important role in lipid and glucose metabolism [6,7]. Many studies have highlighted and documented the lipolytic role of ZAG and its impact on weight reduction, as outlined in this review.

## 2. Materials and Methods

This paper constitutes a literature review of 196 articles available in PubMed (including MEDLINE), Web of Science and Cochrane Library databases. The inclusion criterion for papers was information on the role of zinc and/or ZAG in lipid metabolism. Keywords used to search for papers were “zinc”, “zag”, “zinc-α2-glycoprotein”, “lipids” “lipid metabolism”, “adipose tissue” and “obesity” (Table 1). Experimental studies, randomised trials, clinical trials and review articles were part of the literature subject to the review.

## 3. Zinc and Its Role in the Zinc-α2 Glycoprotein

Zinc (Zn) is currently one of the most important micronutrients in the human body. It is also an essential part of life processes, bone development and body growth.

Zinc plays a major role in the metabolism of carbohydrates, fats and proteins. It is a component of more than three hundred metalloenzymes and exhibits antioxidant activity, thus participating in the reduction of oxidative stress. Zinc is also involved in the synthesis, storage and transport of insulin [3,6,8]. The role and effects of zinc deficiency and excess are shown in Table 2.

Zinc deficiency in the body means that the energy production process is disrupted. This is due to the abnormal behaviour of metalloenzymes that include zinc, such as carbonic anhydrase. It is involved in energy production reactions in the body. When this process is disrupted, fat tissue is formed instead of energy, which promotes the development of overweight and obesity. Studies also show that at low blood levels of this element, lipid management is disrupted leading to an increase in total cholesterol, triglycerides and LDL cholesterol [9,10].

Zinc is a very important regulator of ZAG homeostasis, which plays a role in lipid metabolism and glucose homeostasis [11]. In addition, zinc metabolism changes in obese individuals may result in an impaired ZAG function [12].

ZAG contains trace elements such as zinc. ZAG has been shown to have 2 strong and 15 weak zinc binding sites, and the attachment of zinc at these sites enables ZAG to bind to fatty acids and β-adrenergic receptors. Correct blood zinc concentrations are also essential to maintain adequate ZAG activity, as Zn facilitates the binding of adipokine to substrates. In vitro research has shown zinc binding to play a key role as it induces oligomerisation of the zinc-α2 glycoprotein, allowing ZAG to bind to fatty acids [12,13,14,15].

## 4. Structure and Properties of ZAG

Zinc-α2 glycoprotein is a polypeptide with a molecular weight of 43 kDa. It was first isolated from human plasma in 1961 [16]. ZAG is encoded by the AZGP1 gene. It has been suggested that this gene is a potential obesity gene and may predispose to the development of obesity [17,18]. Glycoprotein has been found in many organs, including the liver, breast, lung and prostate. Recent studies show that ZAG is also present in the adipose tissue of humans, rats and mice [19,20].

The structure of ZAG is similar to major histocompatibility complex (MHC) class 1 proteins. Similar to MHC class I molecules, ZAG has an open groove between its A1 and A2 helix domains. The ZAG groove contains polyethylene glycol (PEG). Calorimetry and fluorescence spectroscopy have shown that the ZAG groove can bind hydrophobic ligands, especially polyunsaturated fatty acids [21,22,23].

ZAG has been classified as a novel adipokine because it is a lipid mobilising factor [24]. Studies have shown that administration of ZAG stimulates lipolysis in isolated fat cells in both mouse and human in vitro and in vivo studies. In addition to lipid mobilisation, ZAG may also regulate the metabolism of excess free fatty acids (FFA) released from adipocytes during enhanced lipolysis [25].

ZAG also affects glucose metabolism and is linked to insulin resistance (IR) [15]. Intravenous administration of ZAG to mice has been shown to lower fasting glucose levels and improve glucose tolerance without changing blood insulin levels 30 min after oral glucose administration [26]. In adult subcutaneous tissue, elevated levels of ZAG play a key role in modulating whole body insulin sensitivity and adipose tissue [27].

ZAG is also used as a tumour marker as its overexpression is found in several types of malignant cancers [28,29,30]. Overexpression of ZAG in cancer results in increased weight loss. On the other hand, the expression of this glycoprotein in obesity is negatively correlated with body weight as well as the amount of body fat in humans and rodents [18,31,32,33].

## 5. Physiological Effects of ZAG

**Figure 1:**
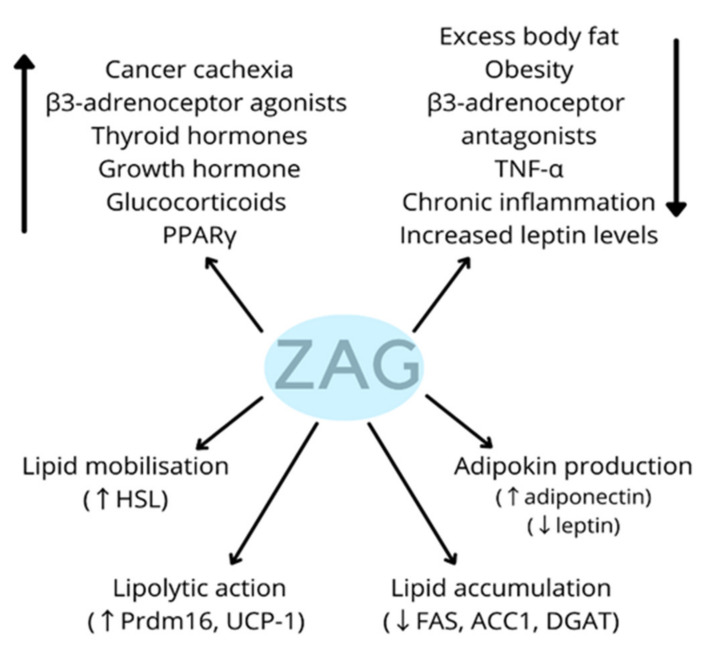
Regulation and role of ZAG. ZAG—Zinc-α2-glycoprotein, PPARγ—peroxisome proliferator-activated receptors γ, TNF-α—tumour necrosis factor-α, HSL—hormone-sensitive lipase, Prdm16—PR domain containing 16, UCP-1—uncoupling protein 1, FAS—fatty acid synthase, ACC1—acetyl-CoA carboxylase, DGAT—diglyceride acyltransferase.

The ZAG impact mechanism on lipid metabolism has not yet been clearly defined, but the overwhelming majority of studies indicate that ZAG may affect this process in multiple ways (Figure 1).

ZAG increases lipolysis in white adipose tissue (WAT) by acting through the classical cyclic AMP pathway [32]. It stimulates the expression of peroxisome proliferator-activated receptor γ (PPARγ) and early B cell factor 2 (EBCF), resulting in increased binding of these molecules to Prdm16 and UCP-1. Prdm16 and UCP-1 promotes white adipose tissue browning and energy consumption, which increases lipolysis [34]. Furthermore, ZAG, through mediating PKA and p38 mitogen-activated protein kinase (MAPK) signalling, can increase the expression of lipolysis-related molecules (UCP-1, PRDM16, CIDEA—cell death activator, PGC-1α—peroxisome proliferator-activated receptor gamma coactivator 1-alpha, NRF-1/2—nuclear respiratory factor 1/2, mtTFA—human mitochondrial transcription factor A, ATGL—adipose triglyceride lipase, HSL, CPT1-A—carnitine palmitoyltransferase I and p-acyl-CoA carboxylase) [35]. The ZAG-induced increase in body temperature and decrease in body weight and body fat can be partly attributed to its effect on UCP-1 in brown adipose tissue, leading to the use of released lipid for heat generation and increased energy expenditure [32,34,36,37]. The lipolytic effects of ZAG have also been attributed to the up-regulation of thermogenin that results from β3-adrenergic receptor activation [24,38].

**Figure 2:**
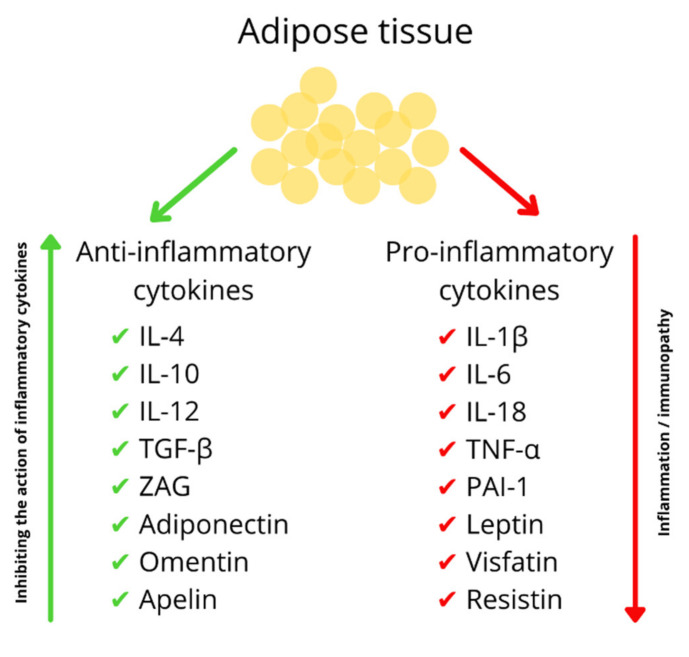
Adipokines produced by the adipose tissue. IL-4—interleukin 4, IL-10—interleukin 10, IL-12 -interleukin 12, TGF-β—transforming growth factor beta, ZAG—zinc-α2-glycoprotein, IL-1β—interleukin 1 beta, IL-6—interleukin 6, IL-18–interleukin 18, TNF-α—tumour necrosis factor-alpha, PAI-1—plasminogen activator inhibitor-1.

Physiologically, ZAG plasma levels range from a few mg/dL in a foetus, 8–12 mg/dL in young people, and 18–30 mg/dL in healthy adults and older men [31]. ZAG is mainly produced by adipocytes, both by visceral and subcutaneous adipose tissue. White adipose tissue is now considered a very important endocrine organ. In addition, it stores triglycerides, which are energy substrates for the body [39,40]. Fat cells produce and secrete proteins called adipokines. It is believed that the adipose tissue of slim people secretes mainly anti-inflammatory cytokines, including adiponectin, ZAG, TGF-β and IL-4. The adipose tissue of obese people, due to the accompanying inflammation, mainly secretes pro-inflammatory cytokines such as: TNF-α, IL-1β, and IL-6 (Figure 2) [41,42,43,44,45]. They act in an autocrine/paracrine manner, affecting nutrient metabolism, modulating appetite, insulin sensitivity and inflammation [46,47,48]. Impaired adipokine secretion can lead to obesity, metabolic syndrome and cardiovascular disease [39].

The expression of ZAG in adipose tissue changes depending on various factors. Increased expression is influenced by PPARγ, glucocorticoids, some β3-adrenergic receptor agonists, thyroid hormones and growth hormone (GH), among others. On the other hand, chronic inflammation and increased serum leptin levels may reduce ZAG secretion in the adipose tissue [12,27,39].

## 6. Impact of Zinc on Lipid Metabolism

Animal studies by Tinkov et al. [49] showed that rats fed a high-fat diet (31.6% fat) had a 34% decrease in zinc levels in adipose tissue compared to rats fed a control diet (with 10% fat). Zinc levels were negatively correlated with leptin, insulin, TNF-α and homeostatic model assessment of insulin resistance (HOMA-IR) values in obese individuals. On the other hand, Charradi et al. [50] in their research observed that a high-fat diet (HFD) in rats induced dyslipidaemia in the blood, caused accumulation of triglycerides and elements such as manganese, copper and zinc, and contributed to decreased lipase activity.

Li et al. [51] in their study described the impact of zinc on hepatic lipid metabolism. Rat hepatocytes were exposed to different concentrations of zinc to investigate the in vitro effect of high zinc levels on lipid synthesis in liver cells. Results indicate that high levels of zinc increase hepatocyte activity and expression of sterol regulatory element-binding transcription factor 1 (SREBP-1), which increase the expression of ACC1, FAS and stearoyl-CoA-1 desaturase (SCD-1) and thereby improve lipid metabolism, while zinc deficiency impairs hepatic lipid metabolism.

A study by Xu et al. [52] suggests that zinc impacts lipid metabolism. Rabbits fed a HFD diet showed significantly abnormal lipids and elevated levels of aspartate aminotransferase (*p* < 0.01) and alanine transaminase (*p* < 0.05). Oral zinc supplementation reversed the effects of the HFD diet. Zinc reduced triglyceride levels and raised HDL-C cholesterol levels, but did not affect changes in total cholesterol and LDL-C cholesterol. In addition, it decreased the expression of high-sensitivity C-reactive protein (hs-CRP) and interleukin-6. The results of these studies suggest that zinc may protect the liver and the cardiovascular function subject to an unhealthy, high-fat diet.

Studies show that people with excessive body fat have reduced levels of zinc and ZAG in their blood [12,53,54,55,56], and its deficiency is a factor in the development of obesity and diabetes [57]. Zinc stimulates lipogenesis and glucose uptake in isolated adipocytes, and zinc ions in the body act as insulin mimetics, affecting the insulin signalling pathway [58].

Studies by Cayir et al. [59] found that low serum zinc concentrations also occur in obese children and adolescents. Blood Zn levels increased with weight loss. An opposite relationship was observed for copper. Cu levels before weight loss were high and decreased as the body mass index (BMI) decreased. This suggests that there is an antagonistic effect between Zn and Cu [60] and that this mechanism may also apply for children.

As shown by Voruganti et al. [61], even short-term (8-week) weight loss in obese women was associated with a 22% increase in serum zinc concentration. Furthermore, it was shown that body zinc content was negatively correlated with body fat percentage.

Findings indicate that impaired Zn homeostasis in obese individuals also affects circulating lipid concentrations in the blood [3]. Numerous studies have found that zinc supplementation significantly reduced blood levels of total cholesterol, LDL cholesterol and triglycerides, potentially reducing cardiovascular morbidity and mortality [62]. Some publications indicate that zinc intake also increases HDL cholesterol levels [63]. Payahoo et al. [64] in their study demonstrated that zinc supplementation affected BMI, body weight and triglyceride levels. One-month administration of zinc gluconate 30 mg/dL to obese subjects resulted in significant reductions in BMI and body weight (35.4 ± 4.3 vs. 34.7 ± 3.9 kg/m^2^, *p* = 0.030 and 90.4 ± 15.4 vs. 88.7 ± 15 kg, *p* = 0.020, respectively) and highly significant reductions in blood triglyceride levels (146.4 ± 6 vs. 131.4 ± 5 mg/dL, *p* = 0.006). On the other hand, in a study by Kelishadi et al. [65], supplementation with zinc at 20 mg/d for 8 weeks not only significantly reduced BMI and Z-score BMI, but also improved lipoprotein profile (decrease in ApoB/ApoA-I ratio, ox-LDL, total cholesterol and LDL cholesterol) and resulted in decreased leptin levels.

Zinc is also involved in regulating the expression of pro-inflammatory cytokines produced by adipocytes [66]. Zinc administration has been shown to positively reduce inflammation in obese individuals with metabolic syndrome [3]. In their study, Jung et al. [67] evaluated the correlation of zinc and inflammatory markers. They found a significant relationship between serum zinc concentrations and levels of IL-6, TNF-α and c-reactive protein (CRP) in women and between zinc and IL-6 levels in men. In conclusion, serum zinc levels were negatively correlated with inflammatory markers. Bao et al. reached similar conclusions [68]. According to analysis, an increase in blood zinc concentration was negatively correlated with the change in inflammatory markers (hs-CRP, MCP-1, VCAM-1 and MDA + HAE) after a 6-month supplementation period of 45 mg zinc per day.

## 7. Impact of ZAG on Lipid Metabolism

Zinc-α2 glycoprotein plays a huge role in the regulation of adipose tissue mass. ZAG acts multidirectionally, playing a role in stimulating lipolysis, inhibiting lipid accumulation in adipose tissue, regulating serum lipid values and influencing the secretion of other adipokines (Table 3).

The results of ongoing studies suggest that ZAG expression in adipocytes is inversely related to fat mass. In a study by Bing et al. [86], it was observed that in mice with a MAC16 tumour, which contributes to the loss of large amounts of adipose tissue, ZAG mRNA and adipokine levels increased tenfold in adipose tissue. In both white adipose tissue and brown adipose tissue (BAT), ZAG content was significantly increased in tumour-bearing mice, indicating that there is a strong association between ZAG levels and the degree of weight loss [47].

Adipocytes and liver cells are major AZGP1 expression sites in rodents [87]. Administration of human recombinant ZAG to ob/ob mice reduces body weight and body fat content without dietary modification [72,88].

Research by Gong et al. [76] clearly shows that serum ZAG levels are significantly lower in obese humans and obese mice, compared to normal-weight test subjects. Glycoprotein levels were also negatively correlated with body weight, BMI, waist circumference, hip circumference, body fat percentage and weight (kg) of body fat. Experiments on KM mice have shown that obese mice fed a high fat diet with established ZAG overexpression experience weight loss without dietary change. The effect of ZAG is correlated with decreased levels of lipogenic enzymes (FAS, ACC1, DGAT) and increased expression of lipolytic enzymes (HSL) in mouse adipose tissue. In addition, in vitro tests showed that incubation of ZAG with adipocytes isolated from mouse adipose tissue stimulates lipolysis to a concentration-dependent extent. This may suggest that ZAG has a direct lipolytic effect.

Fan et al. reached similar conclusions [69]. They found that HSL expression significantly increased in the group of obese mice with ZAG overexpression, compared to obese mice on the control group. Elevated ZAG expression reduced hepatic lipid accumulation in mice with recombinant plasmid and obesity caused by a high-fat diet. In addition, ZAG reduced body weight in mice from the study group.

Furthermore, evidence from Gao et al. [70] indicates that ZAG reduces obesity and improves insulin sensitivity in mice with obesity induced by a high-fat diet. Glycoprotein not only significantly reduced body weight, but also adipose tissue size and adipocyte size in mice. Researchers have shown that this may be due to a reduction in lipid accumulation by ZAG in skeletal muscle.

Russell et al. also observed ZAG impact on adipose tissue browning [71]. Ob/ob mice daily intravenously administered with 100 μg ZAG achieved a 30% reduction in carcass adipose mass and doubled brown adipose tissue mass compared to control mice. In addition to directly inducing lipolysis, ZAG sensitises adipose tissue to other lipolytic stimuli such as HSL and ATGL. The same researchers in other work also observed that ZAG stimulates lipid oxidation in addition to lipid mobilisation. In vitro studies on NMRI and ob/ob mice have shown that ZAG stimulates fatty acid β-oxidation [72,73].

In contrast, Rolli et al. [74] showed that in mice with a deactivated ZAG encoding gene, weight gain was higher after a high-fat diet than in animals with an active ZAG gene. Mice without ZAG also showed reduced lipolysis, even after treatment with lipolysis-inducing agents (FK—forskolin, IBMX—isobutylmethylxanthine, isoprenaline, CL316243).

Studies by Mracek et al. [39] and Ceperuelo-Mallafré et al. [77] found that ZAG may participate in the regulation of adiposes by regulating the products of other adipokines. ZAG expression in human adipose tissue was positively associated with adiponectin expression. Adiponectin has been shown to have anti-inflammatory properties and to increase tissue sensitivity to insulin, which is associated with its decreasing levels in obesity [89,90]. Adiponectin, through activation of AMP kinase, promotes glucose uptake and fatty acid oxidation in skeletal muscle and reduces vascular inflammation [91]. It was initially suggested that the association between ZAG and adiponectin is due to the fact that overexpression of ZAG in 3T3-L1 adipocytes leads to an increase in adiponectin transcripts [17]. Mracek et al. [39] showed that ZAG at physiologically relevant concentrations, increases the release of adiponectin from human adipocytes. This shows that ZAG can stimulate the tissue sensitising effects of insulin and the anti-inflammatory effects of adiponectin. It was also found that there is a negative correlation between ZAG and leptin mRNA levels in visceral and subcutaneous fat in humans. In addition, ZAG mRNA levels were negatively correlated with BMI, fat mass, plasma insulin levels, HOMA-IR model and C-reactive protein. ZAG has been shown to inhibit in vitro leptin production and treatment with recombinant ZAG was found to lead to a reduction in leptin secretion by SGBS cells [86].

White adipose tissue is found in excess in obese and overweight people. Studies indicate that ZAG has the potential to induce WAT browning in 3T3-L1 adipocytes. Xiao et al. [35] observed increased expression of brown adipose tissue-specific genes, such as PRDM16, CIDEA, UCP-1, in adipocytes with ZAG overexpression. Furthermore, ZAG stimulated mitochondrial biogenesis, which is characteristic of adipose tissue browning. An analysis of the results also showed that ZAG could induce lipolysis and inhibit lipogenesis in white adipose tissue as it increased the expression levels of ATGL, HSL, p-HSL and p-ACC lipases. These findings may be used in the future to address obesity and related metabolic disorders [92].

It turns out that ZAG is also found in cord blood and influences the development of body fat as early as infancy. In the study by Näf et al. [78] ZAG in cord blood was positively correlated with fat-free mass, birth weight and gestational age at delivery. On the other hand, Euclydes et al. [79] examined the relationship between ZAG and fat mass in infancy. ZAG concentrations in cord blood were inversely proportional to weight gain in girls. No such relationship was observed for boys’ body fat development.

A study by Yang et al. [80] in a group of men aged between 25–65 years showed that ZAG levels decreased with age. Serum ZAG levels were negatively correlated with waist circumference, BMI, fasting glucose and triglycerides, and positively correlated with total testosterone levels. On the other hand, a study among 258 Chinese by Yeung et al. [81] shows that ZAG concentrations were higher in men than in women (*p* < 0.001) and that they correlated positively with age, waist circumference and BMI, fasting insulin, indices of insulin resistance, adipocyte and fatty acid binding protein (A-FABP), serum TG, and CRP. That research provided the first evidence that the excessive elevation of ZAG in individuals with metabolic disorders and obesity may be a compensatory regulation of the body to the oxidative stress present. Elevated ZAG levels may also indicate ZAG resistance, similar in mechanism to hyperinsulinaemia and hyperleptinaemia caused by the body’s resistance to insulin or leptin. These studies suggest that the serum ZAG concentration value can be considered as a circulating biomarker of obesity and metabolic syndrome. However, further research is needed to determine whether the theses are correct and to fully understand the pathophysiological functions of ZAG.

In contrast, the results obtained by Morse et al. [82] were inconsistent with their hypothesis. They studied the effect of the Roux-En-Y gastric bypass method (RYGB) or very low-calorie diet (VLCD) on changes in ZAG concentrations. After a 12-week intervention, the change in blood ZAG levels was inversely correlated between groups with percentage body fat reduction, weight loss (kg) and percentage weight loss. The reduction in ZAG was significantly greater in the RYGB group, which lost more weight than the VLCD group. The results suggest that ZAG may show a protective effect during more pronounced weight loss. However, the small size of the study group (14 people) limits applicability, so more research is needed to confirm whether ZAG has protective properties.

Liu et al. [75] in their study observed that in addition to reduced serum ZAG levels in obese Chinese people, mRNA levels in subcutaneous white adipose tissue were also reduced. Additionally, they investigated that the role of ZAG in regulating body weight, adipose tissue mass and improving insulin sensitivity in obese mice induced by a high-calorie diet is linked to the activation of WAT browning-related gene expression, specifically PGC1α and UCP1, in subcutaneous WAT and epididymal adipose tissue. These studies reveal a potential role for ZAG in the browning of white adipose tissue.

In addition, Wang et al. [93] examined the relationship between ZAG and the metabolic syndrome (MetS). The study involved 151 patients with MetS, 84 patients with abdominal obesity and 70 healthy controls. Serum ZAG levels were highest in control subjects and decreased with increasing metabolic severity (8.78 ± 1.66 μg/mL for control vs. 8.37 ± 1.52 μg/mL for subjects with central obesity vs. 7.98 ± 0.94 μg/mL for subjects with metabolic syndrome, *p* < 0.05). Reduced ZAG levels were also associated with an increased risk of developing metabolic syndrome (those with the lowest ZAG levels had almost two times the risk of MetS compared to those with the highest ZAG levels). The results of this study suggest that the ratio of serum ZAG to fat mass, may be a future diagnostic biomarker for the diagnosis of metabolic syndrome. Lei et al. reached similar conclusions [94].

In contrast, in the study by Marrades et al. [25], 18 young men were assessed, including 9 with BMI = 23.1 ± 0.4 kg/m^2^ and 9 obese men (BMI = 34.7 ± 1.2 kg/m^2^) with a similar lifestyle. Using RT-PCR, they found that the expression of the ZAG gene was reduced by 70% in the subcutaneous tissue of obese individuals compared to slim subjects. Additionally, analysis of the data showed that gene expression positively correlated with serum adiponectin and negatively correlated with plasma leptin levels and waist circumference in obese subjects.

Overweight and obesity accompany many metabolic diseases, including the polycystic ovary syndrome (PCOS). Studies indicate that ZAG levels are significantly lower in women with PCOS than in healthy women [95,96,97]. Zheng et al. [83] conducted a study to test the effect of ZAG on obesity and the development of insulin resistance in the course of PCOS. 182 women with PCOS and 150 healthy women took part in the tests. Women with PCOS were divided according to BMI or blood glucose levels, of which 82 were overweight or obese. Analysis of the results showed that circulating ZAG levels were significantly lower in women with PCOS than in healthy women (*p* < 0.01). Women with excessive body weight and women with elevated blood glucose levels also exhibited lower blood ZAG levels. It also demonstrated that there was an increase in circulating ZAG after 12 weeks of treatment with either exenatide or metformin (*p* < 0.01). The study shows that ZAG can be used as an observational indicator in the treatment of PCOS.

Using genetic profiling, it was found that ZAG transcripts in adipose tissue are reduced in obese women [98]. It has also been observed that in obese men and women, ZAG gene and protein expression is down-regulated compared to slim individuals [25,77,99]. Reduced ZAG expression in adipocytes during obesity may be associated with impaired adipose tissue metabolism in obesity [86].

ZAG also potentially affects cholesterol metabolism. A study by Olofsson et al. [84] showed that ZAG and total serum cholesterol levels were correlated with each other both in healthy subjects (*p* = 0.00088) and during diet-induced weight loss (*p* = 0.059). Furthermore, in healthy subjects, ZAG levels were correlated with blood triglyceride levels (*p* = 0.035), and in obese subjects on a VLCD diet also with LDL-C (*p* = 0.035 and HDL-C (*p* = 0.055), but not with TG. The ZAG gene was associated with circulating blood cholesterol levels, which may indicate that ZAG plays a role in its metabolism. This correlation may be due to the role ZAG plays in the body, namely in lipolysis. The researchers suggest that the correlation between ZAG and total cholesterol is the result of increased TG lipolysis, caused by the effects of ZAG.

Evidence that ZAG is involved in the development of cancer cachexia was presented by Mracek et al. [85]. In cancer cachexia, there is remodelling of subcutaneous adipose tissue, which assumes the form of shrunken adipocytes with increased fibrosis. Patients with cancer cachexia had a 2.7-fold increase in ZAG mRNA levels, while leptin mRNA levels decreased 2.2-fold. ZAG mRNA was shown to have a positive correlation with weight loss. ZAG release from subcutaneous adipose tissue (SAT) also increased 1.5-fold in cachectic patients and correlated with weight loss. These studies indicate that ZAG expression and secretion is increased in patients with cancer cachexia and that ZAG, as a lipid mobilising factor, may contribute to adipose tissue atrophy.

Both in vivo and in vitro results show that there is increased hormone-induced lipolysis in patients with cancer cachexia [100]. Rydén et al. [33] in their study assessed how ZAG levels change in cancer and obese individuals on a VLCD diet. In vivo tests showed that there was no significant contribution of white adipose tissue to blood ZAG levels. ZAG secreted from WAT, but not its blood levels, correlated positively with poor nutritional status but not with fat mass in patients with gastrointestinal cancer. In obese subjects, WAT-secreted ZAG levels increased significantly, but serum ZAG levels remained unchanged. It was concluded that ZAG is a local factor, and its secretion is activated by the body’s catabolic state and not specifically by cancer cachexia. A model for the regulation of ZAG and fat mass in humans has been proposed. When catabolic processes prevail in the body, e.g., in the course of cancer, cachexia or insufficient food intake, energy-rich lipids are mobilised from adipose tissue by means of lipolysis. This process is initiated by a local increase in ZAG secretion in the adipose tissue. ZAG activates lipolysis, making more free fatty acids available as an energy source.

## 8. Conclusions

There are still too few studies to determine the exact pathway through which zinc and ZAG affect lipid metabolism. Both zinc and ZAG are reduced in overweight and obese individuals, which may indicate that a deficiency of these factors indirectly impacts further development of obesity and associated diseases. Zn and ZAG show a negative correlation with body weight, BMI and adipose tissue. Research also indicates that both zinc and ZAG can regulate the lipid profile, thus preventing the development of cardiovascular diseases. In addition, zinc and ZAG have also been shown to affect other adipokines secreted by the adipose tissue. ZAG expression in human adipose tissue was positively associated with adiponectin expression. Serum zinc levels show a negative correlation with inflammatory markers.

Summarising the currently available studies, it may be concluded that ZAG and Zn should be more widely used as markers for several biochemical reactions occurring in the human body, both in therapeutic and pathogenetic aspects.

## Figures and Tables

**Table 1:** Search strategy.

Objectives	Evaluation of the Existing Literature on the Effects of Zinc and ZAG on Lipid Metabolism Evaluation of the Existing Literature on How Zinc and ZAG Interact on Lipid Metabolism Determining the Current Knowledge
Research question	Does zinc and ZAG have a significant impact on lipid metabolism? Can zinc and ZAG regulate the lipid content in the body?
Keywords	“zinc” or “zag” or “zinc-α2-glycoprotein” and “lipids”; “zinc” or “zag” or “zinc-α2-glycoprotein” and “lipid metabolism”; “zinc” or “zag” or “zinc-α2-glycoprotein” and “adipose tissue”; “zinc” or “zag” or “zinc-α2-glycoprotein” and “obesity”.

**Table 2:** Role of zinc in the human body and the effects of its deficiency and excess.

Role	component of many proteins and enzymes affects the regulation of the acid-base balance takes part in replication, transcription and expression of genes through RNA and DNA polymerases is involved in bone mineralization and wound healing guarantees the proper functioning of the pancreas supports the work of the immune system maintaining a healthy body weight
Deficiency	*acrodermatis enteropathica* diarrhea loss of appetite alopecia hypogonadism infertility growth retardation reduced immunity impaired wound healing infect blindness mental disorders may promote atherosclerosis may promote weight gain
Excess	lethargy focal neuronal deficits nausea/vomiting epigastric pain diarrhea elevated risk od prostate cancer copper deficiency altered lymphocyte function decreased immunity decreased concentration of HDL cholesterol

**Table 3:** Impact of ZAG on lipid metabolism.

Type of Study	Type of Cells/ Rodents/ Participants	Country	Assessment	Results	Reference
In vitro cells	Mouse 3T3-L1 fibroblast cells	China	Cells were cultured at 37 °C in 5% CO_2_ in DMEM and 25 mM glucose and 10% FBS. 3T3-L1 adipocytes were fixed in 10% formalin. Stained cells in Oil Red O. 3T3-L1 adipocytes—examined under a transmission electron microscope, immunofluorescence, Quantitative real-time PCR, Western blot analysis.	- ZAG induces brown-like changes in adipocytes (*p* > 0.05). - ZAG stimulates mitochondrial biogenesis in 3T3-L1 adipocytes (*p* > 0.05). - ZAG promotes lipid metabolism in adipocytes (*p* > 0.05). - ZAG-induced browning programme is mediated through PKA and p38 MAPK signaling (*p* > 0.05).	Xiao et al. [35]
In vitro cells	Adipose tissue (visceral and subcutaneous) was collected from human subjects with a wide range of BMIs.	United Kingdom	ZAG mRNA levels were quantified by real-time PCR and protein by Western blotting.	- ZAG mRNA level was negatively correlated with BMI (*p* < 0.001)*,* and fat mass (*p* < 0.01, visceral; *p* < 0.05, subcutaneous). - ZAG mRNA was positively correlated with adiponectin (*p* < 0.05, visceral; *p* < 0.001, subcutaneous) but negatively associated with leptin mRNA (*p* < 0.05, visceral; *p* < 0.05, subcutaneous).	Mracek et al. [39]
Animal	32 male specific pathogen-free (SPF) mice *n* = 16—normal diet (ND) *n* = 16—high-fat diet (HF)	China	Injection of ZAG recombinant plasmid. The blood and liver samples. Hepatic lipid accumulation was evaluated by Oil Red O staining. RNA isolation, quantitative real-time PCR and Western blotting was conducted.	- Decreased body weight (*p* < 0.05), hepatic TG (*p* < 0.01), stearoyl-CoA desaturase-1 (SCD1) and Acyl-CoA Synthetase-1 (ACSS1) protein levels (*p* < 0.05). - Increased hormone sensitive lipase (P-HSL) levels (*p* < 0.05).	Fan et al. [69]
Animal	Four-week-old specific pathogen-free male mice; three groups: normal diet, high-fat diet (HFD), and ZAG treatment under HFD (HFZ).	China	Blood and tissue samples. Biochemical analysis and glycogen assay, RNA isolation and quantitative real-time PCR analysis were conducted. Protein concentration was tested by Western blot analysis. HSL concentrations were assayed using an enzyme-linked immunosorbent assay kit Glucose and insulin tolerance tests.	- ZAG treatment observably reduced body weight and decreased the size of the fat mass and adipose cells (*p* < 0.05). - Epididymal fat weight and perinephric fat weight were significantly reduced (*p* < 0.05). - Insulin sensitivityof ZAG-treated mice was significantly improved (*p* < 0.05). - Concentrationsof HSL increased by the ZAG treatment (*p* < 0.05).	Gao et al. [70]
Animal	Obese hyperglycaemic (ob/ob) mice	United Kingdom	Treatment animals for 15 days with ZAG (100 mg, intravenously, daily). Blood—Western blot analysisAdipocytes—Lipolytic assay, Lipolysis in vivo	Treatment with ZAG caused: - reduction of body weight (*p* < 0.001), - reduction in carcass fat mass, - increase in weight of brown adipose tissue, - increased expression of ZAG and hormone-sensitive lipase (HSL).	Russell et al. [71]
Animal	Homozygous obese (ob/ob) male mice	United Kingdom	Adipose cells were cultured. ZAG was treated with purification. ZAG was administered (50 µg in 100 µL PBS) daily by iv administration, whereas the control group received an equal volume of PBS, and body weight and food and water intake were monitored daily. The pancreas was removed and its insulin content was examined by using a mouse insulin ELISA kit. Lipid oxidation and accumulation and Western blot analysis was conducted.	- Rise of body temperature (*p* < 0.001). - Blood glucose levels were reduced (*p* < 0.001). - Increased rate of oxidation of the triolein during the 24-h study, with overall lipid oxidation 36% (*p* < 0.05). - Increased the expression of UCP1, UCP3 in BAT as well as UCP3 in skeletal muscle (*p* < 0.001).	Russell et al. [72]
Animal	Ex-breeder male NMRI mice	United Kingdom	Lipid mobilising factor (LMF) was purified from the urine of weight losing patients with pancreatic cancer. LMF (8 mg in 100 mL PBS) was administered b.d. by i.v. administation into the tail vein of ex-breeder male NMRI mice. Studies of glucose use and lipid oxidation and accumulation were conducted.	- Increase lipid accumulation in plasma, liver and white and brown adipose tissue after administration of lipid mobilising factor (*p* < 0.05). - Increased overall lipid oxidation (*p* < 0.05). - Tissue glucose metabolic rate increased almost threefold in brain (*p* < 0.05).	Russell et al. [73]
Animal	Wild-type mice	France	Gene targeting in embryonic stem cells and generation of ZAG deficient mice. Sub-confluent cells were treated with tunicamycin and then deglycosylated. Epididymal adipose tissue was performed by lipolytic assay. Real-Time RT-PCR was conducted.	- Weight gain in mice lacking the ZAG gene (*p* > 0.05). - Reduced lipolysis in mice lacking the ZAG gene (*p* > 0.05).	Rolli et al. [74]
Animal	31 eight-week-old male ICR mice divided into standard food diet (SF) group (n = 10) and high-fat diet HFD group (n = 21)	China	Intraperitoneal insulin tolerance test and Intraperitoneal glucose tolerance test were conducted. Tissue samples and measurements of biochemical parameters were taken. Adipose tissue was morphological and immunohistochemical stained. RNA isolation and reverse transcription quantitative PCR (RT-qPCR) analysis and Wester Blot analysis were conducted. ZAG expression plasmid (5 μg/injection, four times a week) was injected in HFD-induced obese mice for 8 weeks.	- Reduction in body weight was observed at 6 weeks after ZAG treatment (*p* < 0.05). - ZAG overexpression significantly decreased body weight and WAT mass, and greatly increased the glucose tolerance of obese mice, as shown by the intraperitoneal glucose tolerance test and intraperitoneal insulin tolerance test (*p* < 0.05). - Positive correlation between the expression levels of Zag and Pgc1α in mouse sWAT (*p* < 0.05). - mRNA levels of Pgc1a, glucose- 6-phosphatase, catalytic (G6pc), phosphoenolpyruvate carboxykinase 1, cytosolic (Pck1) and glycogen synthase 2 (Gys2) in the liver tissue of HFD-induced obese mice were significantly decreased (*p* < 0.05).	Liu et al. [75]
Animal	36 Male, obese kunming (KM) mice inducted by high-fat diet (HFD)	China	Animal in vivo plasmid DNA transfecting, Western blotting for assays of serum ZAG level in mice and Real-time fluorescence quantitative PCR analysis for fatty metabolic enzymes expressions in mice adipose tissue was performed.	- ZAG level was significantly lower in obese patients and obese mice in comparison to that in people and mice with normal weight. - ZAG overexpression in mice reduced body weight and the percentage of epididymal fat. - The decreased FAS, ACC1 and DGAT mRNA and the increased HSL mRNA were also observed in epididymal adipose tissue in ZAG overexpression mice.	Gong et al. [76]
Human	28 overweight or obese male and female (BMI ≥ 24 kg/m^2^) and 16 normal-sized control male and female (BMI < 24 kg/m^2^)	China	Blood and urine tests. Body weight, height, body mass index (BMI), percentage of body fat (fat %), fat mass, free fat mass (FFM) and total body water (TBW)—bioelectrical impedance analyser. Serum ZAG level was determined by commercially available human zinc-alpha2-glycoprotein ELISA kit.	- ZAG level was significantly lower in obese patients and obese mice in comparison to that in people and mice with normal weight. - ZAG level was negatively correlated with body weight (*p* < 0.001), body mass index (*p* < 0.001), waist circumference (*p* < 0.001), hip circumference (*p* < 0.001), percentage of body fat (*p* < 0.03) and fat mass (*p* < 0.01).	Gong et al. [76]
Human	73 Caucasian (43 male and 30 female)	Spain	Plasma and adipose tissue [sc (SAT) and visceral (VAT)]. mRNA of PPARγ, hormone-sensitive lipase (HSL), adipose triglyceride lipase, adiponectin, omentin, visfatin, and ZAG were quantified. Plasma concentrations of ZAG were determined with ELISA.	- ZAG plasma levels showed a negative correlation with insulin (*p* < 0.008) and the HOMA- IR for insulin resistance index (*p* < 0.016). - ZAG expression in SAT was reduced in overweight and obese individuals compared with lean subjects (*p* < 0.001 and *p* < 0.007). - SAT ZAG was predicted by adiponectin mRNA expression (*p* < 0.0001) and plasma triglyceride levels (*p* < 0.006).	Ceperuelo-Mallafre et al. [77]
Human	207 pregnant women (130 with normal glucose tolerance (NGT) and 77 with GDM)	Spain	Women were recruited in the early third trimester and their offspring were studied. Cord blood was obtained at delivery and neonatal anthropometry was assessed in the first 48 h. ZAG was determined in maternal serum and cord blood.	- ZAG concentration was lower in cord blood than in maternal serum (*p* < 0.001). - Serum mZAG concentrations showed a positive correlation with HDL cholesterol levels and a negative correlation with triglyceride levels, insulin and HOMA-IR - positive correlation between maternal ZAG and maternal adiponectin levels (*p* = 0.003). - cord blood ZAG (cbZAG) was positively correlated with fat-free mass, birth weight and gestational age at delivery (*p* < 0.001).	Näf et al. [78]
Human	104 mother–infant pairs	Brazil	Cord blood leptin, ZAG, and adiponectin—by enzyme-linked immunosorbent assays. The body composition of the infants—monthly by air displacement plethysmography. A multiple linear regression analysis was conducted with the average fat mass gain from birth to the third month of life as the outcome and cord blood leptin, ZAG, and adiponectin as the variables.	- Leptin was inversely associated with fat mass gain in the first 3 mo of life (*p* = 0.003). - There were inverse associations of leptin (*p* = 0.021), ZAG (*p* = 0.042), and maternal body mass index (*p* = 0.04) with fat mass gain in girls but fat mass gain in boys was positively associated with gestational age (*p* = 0.01).	Euclydes et al. [79]
Human	297 men aged 25–65 years, 152 with hyperlipaemia (HL) and the other 145 with normal blood lipid (normal control). They were divided into four age groups (25–35 yr, 36–45 yr, 46–55 yr, and 56–65 yr) and three tertile groups (Q1, Q2, and Q3) according to the tertiles of the serum ZAG level	China	Blood lipid, blood glucose, serum ZAG, and reproductive hormones	- The serum ZAG level decreased gradually with the increase of age in both the HL patients and normal controls, significantly in the 36–45 and 56–65 yr age groups (*p* < 0.05) and lower in the HL than in the control men in the 25–35 and 36–45 yr groups (*p* < 0.05). - The levels of follicle-stimulating hormone (FSH) and total testosterone (TT) changed significantly with the ZAG level. - The level of serum ZAG was correlated negatively with age (*p* < 0.05), waist circumference (*p* < 0.05), body mass index (BMI) (*p* < 0.05), fasting blood glucose (*p* < 0.05), and triglyceride (TG) (*p* < 0.05). - The level of serum ZAG was correlated positively with TT (*p* < 0.05). - Age, BMI and TG were independent factors influencing the serum ZAG level.	Yang et al. [80]
Human	A total of 258 Chinese participants (aged 55.1 ± 12.5 yr; 120 males, 138 females; body mass index (BMI), 25.4 ± 4.1 kg/m^2^)	China	Serum ZAG levels— determined with ELISA. The relationship between serum ZAG levels and cardiometabolic parameters was assessed.	- Serum ZAG levels were higher in men (*p* < 0.001 vs. women). - Serum ZAG correlated positively with age, parameters of adiposity (waist circumference and BMI), fasting insulin, insulin resistance indices, serum triglycerides, adipocyte-fatty acid-binding protein, and C-reactive protein, and diastolic blood pressure (all *p* < 0.005, age- and sex-adjusted), and inversely with highdensity lipoprotein-cholesterol levels (*p* < 0.008, age- and sex-adjusted). - Elevated progressivelywith an increasing number of components of the metabolic syndrome (*p* < 0.001).	Yeung et al. [81]
Human	14 healthy, obese individuals (ages 18 and 65, had a BMI between 35 and 50 kg/m^2^) underwent either RYGB (N = 6) surgery or a very low calorie diet (VLCD) (N = 8)	USA	Body composition and fasting plasma ZAG concentrations were measured at baseline (pre) and 12 weeks post intervention (post). Blood tests ZAG—ELISA kit	- No difference in plasma ZAG between the two intervention groups pre-intervention. - Post-intervention, there was a significant overall reduction (*p* < 0.001) in plasma ZAG, which was significant only within the RYGB group from pre to post intervention (*p* < 0.015) and significantly greater than the change within the VLCD group. - ZAG was correlated across groups with BMI reduction (*p* < 0.05), % body fat reduction (*p* < 0.015), reduction in weight (*p* < 0.05), and % weight loss (*p* < 0.05). - ZAG may have a protective effect during marked weight loss.	Morse et al. [82]
Human	40 overweight/obese patients (BMI ≥ 24 kg/m^2^, age 42.8 ± 4.5 yr) and 40 lean control participants (BMI < 24 kg/m^2^, age 44.6± 8.3 yr)	China	Physical and clinical examinations. Blood samples. Fasting insulin was measured by a Siemens Centaur XP system. ZAG—enzyme-linked immunosorbent assay (ELISA) kits. Human sWAT tissue was collected by laparoscopic gastric surgery. RNA was isolated from sWAT by use of an E.Z.N.A.	- Serum ZAG levels were negatively correlated with BMI, body weight and fat mass after adjusting for age and sex in all subjects (*p* < 0.05). - ZAG mRNA expression in the sWAT of obese patients was significantly decreased (*p* < 0.01) - Positive relationship in mRNA levels between ZAG and WAT browning related genes, including UCP1, PGC1α, PRDM16, CIDEA, and PPARγ2 after adjustment for age, sex and BMI (all *p* < 0.05).	Liu et al. [75]
Human	151 MetS patients, 84 patients with central obesity and 70 healthy controls	China	General clinical information, serum samples were obtained from all subjects and serum ZAG levels were determined via the commercial ELISA kits.	- Serum ZAG levels were the highest in the control group, then gradually decreased with the severity of the metabolic abnormalities increased (*p* < 0.05). - Serum ZAG levels decreased progressively with an increasing number of the MetS components (*p* for trend = 0.002). - Subjects with the highest tertile of ZAG, subjects in the lowest tertile of ZAG had 1.946-fold higher risk of MetS (*p* = 0.004).	Wang et al. [46]
Human	18 young men, 9 lean (BMI = 23.1 ± 0.4 kg/m^2^) and 9 obese (34.7 ± 1.2 kg/m^2^)	Spain	ZAG expression was determined by real-time PCR analysis in subcutaneous abdominal adipose tissue	- ZAG gene was downregulated in subcutaneous adipose tissue of obese compared to lean subjects (*p* < 0.05) - Positive correlations between ZAG gene expression and serum adiponectin (*p* < 0.01) and a negative correlation with the plasma levels of leptin (*p* < 0.05) and waist circumference (*p* < 0.05) were found in obese subjects.	Marrades et al. [25]
Human	182 patients 18 to 40 years with PCOS; 150 controls without PCOS (18 to 40 years old)	China	Women with PCOS were partitioned into groups according to body mass index or blood glucose concentrations, determined serum ZAG, anthropometric parameters, metabolic and endocrine indicators, and inflammatory markers. 82 overweight/obese subjects of the recruited women with PCOS were randomly assigned to groups administered either 12 weeks of exenatide injection (10 μg b.i.d.) or oral metformin (1000 mg b.i.d.). Circulating ZAG levels were determined after 12 weeks of treatment by enzyme-linked immunosorbent assay.	- Circulating ZAG was significantly lower in PCOS women than in healthy women (*p* < 0.01). - Overweight/obese women and those with higher blood glucose levels had lower circulating ZAG. - After 12 weeks of exenatide or metformin treatment, there were significant increases (*p* < 0.01) in circulating ZAG in both treatment groups (both *p* < 0.01).	Zheng et al. [83]
Human	186 healthy participants were selected for genotyping; 228 subjects for analysing serum ZAG; the ZAG levels were also analysed in 62 obese subjects before, during, and 2 weeks after a very low calorie diet (VLCD, 450 kcal/d for 16 weeks)	Sweden	Serum ZAG concentrations—an inhouse immunoassay and enzyme-linked immunosorbent assay	- Serum levels of ZAG correlated with serum levels of cholesterol (*p =* 0.00088) in healthy subjects and during weight loss (*p* = 0.059). - ZAG genotype was associated with total cholesterol (*p* = 0.014) and low-density lipoprotein cholesterol (*p* = 0.026) in healthy subjects	Olofsson et al. [84]
Human	I study: 8 weight-stable and 17 cachectic cancer patients II study: 18 weight-stable and 15 cachectic cancer patients	United Kingdom	Zinc-α2-glycoprotein mRNA and protein expression were assessed in subcutaneous adipose tissue (SAT), subcutaneous adipose tissue morphology was examined and serum ZAG concentrations were quantified. The effect of ZAG on lipolysis was evaluated In vitro.	- ZAG mRNA was upregulated (2.7-fold, *p* < 0.028) while leptin mRNA decreased (2.2-fold, *p* < 0.018). - ZAG mRNA correlated positively with weight loss (*p* < 0.01) and serum glycerol levels (*p* < 0.003). - Zinc-α2-glycoprotein release by SAT was also elevated in cachectic patients (1.5-fold, *p* < 0.024) and correlated with weight loss (*p* < 0.003). - Recombinant ZAG stimulated lipolysis in humanadipocytes.	Mracek et al. [85]
Human	1 cohort—4 participants undergoing cosmetic liposuction; 2 cohort—10 (seven men and three women) otherwise healthy participants with a large range of age (27–70 years) and BMI values (23–45 kg/m^2^); 3 cohort—34 patients with newly diagnosed gastrointestinal cancer; 4 cohort—10 obese but otherwise healthy women subjected to caloric restriction with a VLCD	Sweden	ZAG levels in serum and in conditioned medium from WAT⁄ adipocytes—by enzyme-linked immunosorbent assay. ZAG release from WAT in vivo was determined in 10 healthy participants. Adipose tissue—fat biopsies, isolation of adipocytes from adipose tissue. Measurements of glycerol, nonesterified fatty acids and ZAG.	- ZAG was released from abdominal WAT and adipocytes in vitro (*p* < 0.05). - Secretion of ZAG from adipose tissue, but not serum levels, correlates with nutritional status in patients with cancer (*p* < 0.001). - Significant and strong positive correlation between percentage weight loss and the increase in adipose ZAG secretion (*p* = 0.019). - In obese subjects on a VLCD, ZAG secretion from WAT increased significantly whereas serum levels remained unaltered.	Rydén et al. [33]
